# Optimizing the Treatment of Recycled Aggregate (>4 mm), Artificial Intelligence and Analytical Approaches

**DOI:** 10.3390/ma16082994

**Published:** 2023-04-10

**Authors:** Hasan Dilbas

**Affiliations:** Civil Engineering Department, Engineering Faculty, Van Yuzuncu Yil University, 65080 Van, Turkey; hasandilbas@yyu.edu.tr; Tel.: +90-444-50-65

**Keywords:** recycled aggregate, ball mill method, optimization, abrasion coefficient, artificial neural networks

## Abstract

Attached, old mortar removal methods are evolving to improve recycled aggregate quality. Despite the improved quality of recycled aggregate, treatment of recycled aggregate at the required level cannot be obtained and predicted well. In the present study, an analytical approach was developed and proposed to use the Ball Mill Method smartly. As a result, more interesting and unique results were found. One of the interesting results was the abrasion coefficient which was composed according to experimental test results; and the Abrasion Coefficient enables quick decision-making to get the best results for recycled aggregate before the Ball mill method application on recycled aggregate. The proposed approach provided an adjustment in water absorption of recycled aggregate, and the required reduction level in water absorption of recycled aggregate was easily achieved by accurately composing Ball Mill Method combinations (drum rotation-steel ball). In addition, artificial neural network models were built for the Ball Mill Method The artificial neural network input parameters were Ball Mill Method drum rotations, steel ball numbers and/or Abrasion Coefficient, and the output parameter was the water absorption of recycled aggregate. Training and testing processes were conducted using the Ball Mill Method results, and the results were compared with test data. Eventually, the developed approach gave the Ball Mill Method more ability and more effectiveness. Also, the predicted results of the proposed Abrasion Coefficient were found close to the experimental and literature data. Besides, an artificial neural network was found to be a useful tool for the prediction of water absorption of processed recycled aggregate.

## 1. Introduction

Environmental concerns nowadays occupy countries’ agendas, and laws have been enacted to struggle with waste. Waste disposal through recycling has been the main subject of the measures, and governments pondered environmentally friendly implementations in waste recycling. In the structural industry, the green building concept encourages the use of construction and demolition waste (CDW). Waste recycling in green structures may be an effective way to reduce the amount of CDW. RA is a byproduct obtained as a result of CDW in aggregate size (usually in the range of 0.125–32 mm). There is a high amount of waste concrete in the content of CDW, and in addition to this, it may also contain other content, such as plaster, marble, and tile. However, the use of CDW consisting of waste concrete as RA is more common, and its properties are better than others. Due to the presence of AOM in RA, the usability of RA in possible engineering applications is limited [1,2]. Research on concrete, including RA (RAC), reported that the lower the strength, elasticity modulus and density, the higher the water absorption of RA due to attached old mortar (AOM) affected the properties of concrete unfavorably [3,4,5,6,7,8,9,10,11,12]. Therefore, researchers aimed to improve the low physical and mechanical properties of RA compared to those of NA and also to make use of RA feasible in the production of concrete. Hence, numerous methods were proposed for the “removal of AOM” and “strengthening AOM” [13]. The suggested processes to strengthen AOM and remove AOM considered the lower strength capacity, the higher porosity, and the higher water absorption properties of RA to improve the quality of RA. Pozzolan slurry emulsion and polymer emulsion, cement/bio-cement treatment, bio-deposition treatment, carbonation treatment, acid treatment (i.e., 0.1 M HCl), heat treatment, microwave treatment, and also the combination of the treatment methods were applied to RA for the “removal of AOM” and “strengthening AOM” [1,13,14,15,16,17,18,19,20]. However, there was a gap in the related subjects, and it was that in the studies conducted, researchers only examined the treatment methods and their effectiveness on RA. In addition, they rarely analyzed the gradual improvement in RA after applying the improvement method analytically and with artificial intelligence. Also, they had not fully considered the extent to which the RA feature would be healed and at which level the healing method was applied. Although there were some studies on determining the most effective improvement step for mechanical improvement (i.e., [14,21,22,23,24]), the situation described was not properly considered in the studies.

CO_2_ emission, energy consumption, and especially harmful effects on the natural environment of the treatment methods are important topics, and these should be considered before use [25]. In this regard, mechanical abrasion treatment is one of the successful methods in consideration of the required energy consumption and its impact on nature [25]. The Ball Mill Method (BMM) is a mechanical abrasion treatment and has been used alone (i.e., [23]) or with a treatment method (i.e., heat treatment [19], silica fume [26]) in the literature. In addition, the application of the treatment methods to the recycling industry showed that grinding and mechanical treatment might be found more feasible than others because the environmental impact of the mechanical treatment is lower than other methods [25]. However, the gradual improvement effect of BMM on RA was rarely studied in terms of analytical and/or artificial intelligence approaches.

The aim of this study was to close the gaps in the literature mentioned above with substantial outcomes on the use of RA in a fruitful systematic way for the researchers and the concrete industry. Hence, first, a theoretical study was conducted to define how BMM reduced water absorption of RA depending on literature data and a formula was composed to predict water absorption of RA processed by BMM. Then, second, the formula was generalized for RAs sourced by low, medium, and high-strength concrete waste defining the “abrasion coefficient” term in this systematic study. Third, artificial neural network (ANNs) models for BMM were constructed in MATLAB. Then, ANN models were trained and tested.

## 2. Materials and Method

### 2.1. Materials

In the literature, optimization processes of the Ball Mill Method (BMM) on 4–11.2 mm and 11.2–22.4 mm RAs were studied regarding standard Los Angeles test equipment and the water absorption values of RA sourced by the low-strength concrete waste (CW) before and after various BMM applications (Table 1, Table 2 and Table 3) [21]. In Table 2 and Table 3, the combinations, including steel balls (S) and drum rotations (R), are given, and the water absorption (WA) values of each processed RAs are presented [21]. The data are used in this paper in a theoretical approach.

It was reported that when RA is required in concrete, if the aggregate, which makes up approximately 70% of the concrete, has a high-water absorption value, it disrupts the “effective water/binder ratio” of the concrete and, therefore the concrete may experience strength and durability problems [27]. In order to obtain sufficient concrete strength/strength, the problem of the high-WA value of RA compared to NA needs to be solved. The high water absorption value of RA compared to NA is due to the AOM content [28]. AOM is a porous structure with cracked, fissured cavities. The RA remediation effort also has this aim (e.g., BMM). For this reason, only the WA of RA was taken into account in this study, and the development of a solution using a BMM–artificial intelligence–analytical approach in order to obtain the desired RA water absorption values is examined in the article.

According to the data used in this paper, given in Ref. [21], the source quality of RA was of low-strength quality (<20 MPa), and the source had a high water absorption value (10.11%). The low-quality sourced RA was treated by BMM with 100 to 500 drum rotations and 2 to 12 standard Los Angeles abrasion machine steel balls (here, each steel ball weighed 400 to 445 g). However, the treatment was adaptable with respect to other RA sizes, such as 4–11.2 mm and 11.2–22.4 mm. Thus, the detailed treatment process was applied to the RA, and the treatment grade can be observed easily. Here, the processed RAs are tested, and the water absorption properties are determined. The water absorption test results are the average of the three test results, as given in Table 1, Table 2 and Table 3.

### 2.2. Methodology

#### 2.2.1. Equation Derivation for BMM

It can be observed from Table 2 and Table 3 that increasing the number of R–S decreased the WA of RA in general, and the data seemed to indicate that the values were a function of R and S. The relationship between WA, R and S was analyzed by using MATLAB 2020a (MATrix LABoratory), defining the data in a matrix form (Equation (1)).

(1) $$\left[\begin{array}{ccc} {WA}_{11} & \cdots & {WA}_{1n}\\ \vdots & \ddots & \vdots \\ {WA}_{n1} & \cdots & {WA}_{nn} \end{array}\right]=a\times \left[\begin{array}{ccc} R_{11} & \cdots & R_{1n}\\ \vdots & \ddots & \vdots \\ R_{n1} & \cdots & R_{nn} \end{array}\right]+b\times \left[\begin{array}{ccc} S_{11} & \cdots & S_{1n}\\ \vdots & \ddots & \vdots \\ S_{n1} & \cdots & S_{nn} \end{array}\right]$$

here, WA, R and S were matrix elements. WA is the water absorption value relative to the initial water absorption, R and S are the numbers of drum rotations and steel balls, and a and b are coefficients. The general form of the derivation is presented in Equation (2).

WA = a × R^n^ + b × S^m^ (2)

where a and b are coefficients, WA is the water absorption value relative to initial water absorption, and R and S are the numbers of drum rotations and steel balls. Then, a relationship is found with a great accuracy/correlation coefficient (Table 4).

#### 2.2.2. Abrasion Coefficient for BMM

BMM can be defined as a function of three main parameters, such as no. of R and S, and water absorption of RA, and this situation caused difficulty in the evaluation of the result. Hence, a systematical parameter elimination work was conducted to examine how many S is equal to R. According to this, the number of “steel ball equivalence” (SBE) was found, and then the “Abrasion Coefficient” (AC) was defined.

In the process, at first, the relations between R and S were described and examined. Approximately the same water absorptions of various R-S combinations were obtained using the best-fit equations given in Table 4 (Table 5 and Table 6). Also, it was assumed that a linear relation between R and S was there while the change in the number of R and S caused approximately the same water absorption. This phenomenon was investigated as follows:

R = c × S(3)

(4) $$C=\sum_{i=1}^{n}\frac{c_{i}}{n}$$

where R and S are the numbers of drum rotations and steel balls, c and C are coefficients, and C is the average of c. Also, here C shows the SBE.

After various iterations, C was found to be approximately 42 for RAs and is independent of the aggregate size, such as 4–11.2 mm and 11.2–22.4 mm. Then, the Abrasion Coefficient (AC) equation was formed to define the BMM combination impact on RA (Equation (5)).

**Figure 1:**
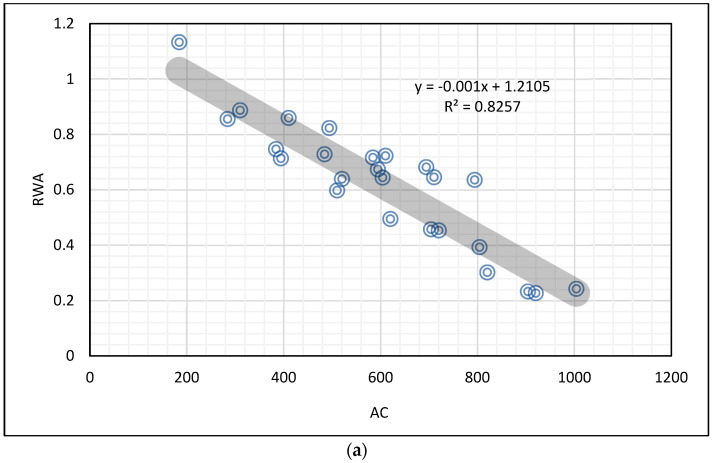

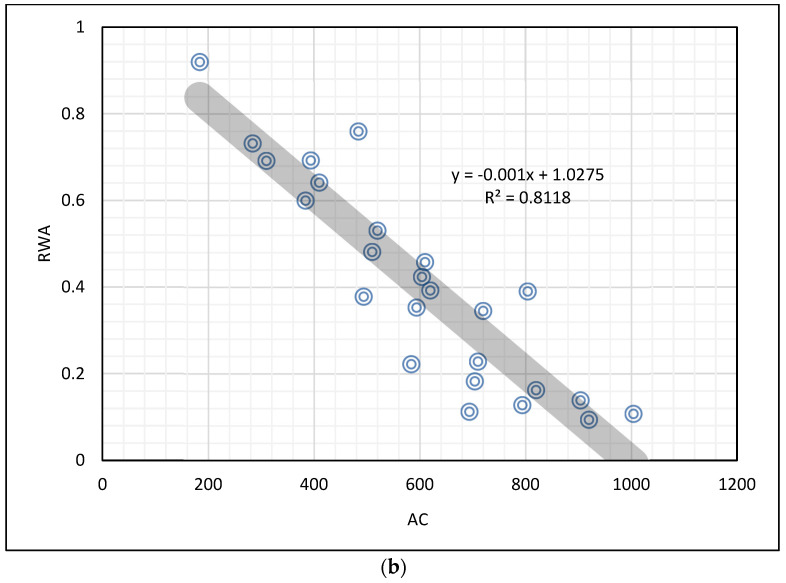
Relative water absorption and Abrasion Coefficient relation for (**a**) RA with 4.0-11.2 mm (**b**) RA with 11.2-22.4 mm.

AC = R + 42 × S (5)

where AC is the Abrasion Coefficient, R and S are the numbers of rotations and steel balls, and the MSE value of Equation (5) is 23.83%. The relation between AC and the relative WA (RWA) of RA is given in Figure 1, and Equation (6) was determined for AC and RWA. Here, the RWA of RA was relative to the initial WA of RA. In this paper, the maximum aggregate size is considered to be 22.4 mm, and hence the equation obtained in Figure 1 (Equations (6) and (7)) found RAs in sizes of up to 22.4 mm.

RWA = −0.001 (AC) + 1.2105(6)

RWA = −0.001 (AC) + 1.0275(7)

where RWA is the ratio of the water absorption of processed RA to the initial water absorption of RA, and AC is the Abrasion Coefficient calculated according to Equation (5). In addition, the MSE of Equations (6) and (7) are calculated as 14.39% and 33.26%, respectively. According to Equation (6), the required reduction in water absorption of RA could be determined by adjusting R-S in the BMM combinations, and Equation (6) eases composing an optimum BMM combination to obtain the required quality level for RA having a minimum process time.

#### 2.2.3. Artificial Neural Network Model for BMM

**Figure 2:**
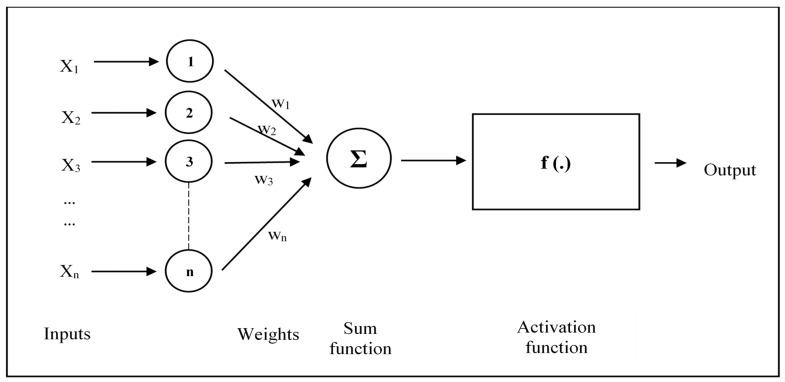
Artificial neuron network model.

**Figure 3:**
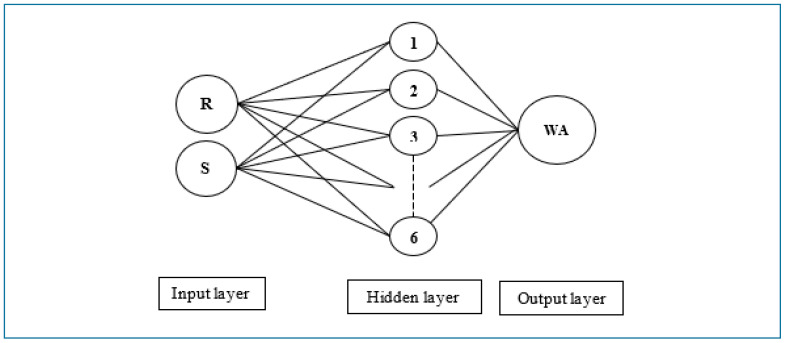
Artificial neuron network architecture for drum rotation-steel ball-water absorption.

**Figure 4:**
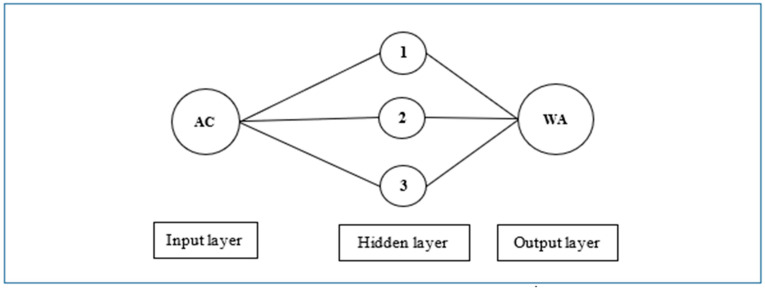
Artificial neuron network architecture for Abrasion Coefficient-water absorption of recycled aggregate.

**Figure 5:**
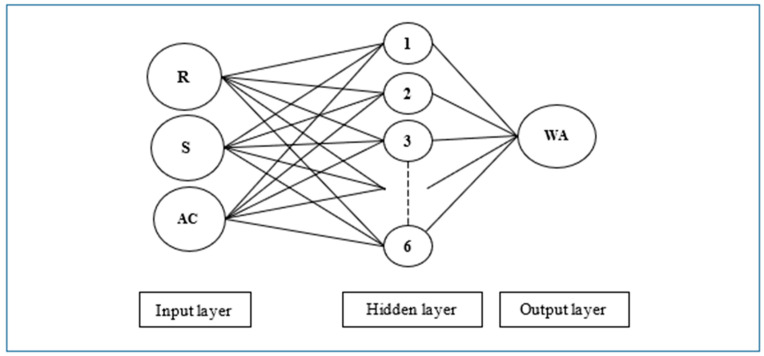
Artificial neuron network architecture for rotation-steel ball-Abrasion-Coefficient-water absorption.

Artificial neural networks (ANN) imitate the brain of humans and include simple processing elements named neurons. Figure 2 presents an artificial neuron model and shows the components. X_n_ presents the inputs to the model, and w_j_ are the weights for each X_j_ input. In total, 60 BMM combinations were employed and included 100–200–300–400–500 drum rotations and 0–2–5–7–10–12 steel balls for two RA sizes. In total, 180 parameters, such as 60 drum rotations, 60 steel ball numbers and 60 Abrasion Coefficients, were utilized to build R-S-WA, AC-WA, and R-S-AC-WA ANN models (Figure 3, Figure 4 and Figure 5). The water absorption of RA processed by BMM considering 60 combinations (30 + 30 for 4.0–11.2 mm and 11.2–22.4 mm RAs, respectively, see Table 2 and Table 3) are the only output values in this research. The Abrasion Coefficients of the combinations were calculated by using Equation (6).

Three ANN architectures were built for the relationships, such as R-S, AC and R-S-AC. The first architecture had a 2-6-1 model and was built for R-S-WA. The second architecture had a 1-3-1 model and was built for AC-WA. The third architecture had a 3-6-1 model and was built for AC-WA parameters. The architectures are shown in Figure 3, Figure 4 and Figure 5. It should be noted that the trial and error method was used in the development of the ANNs, and the presented ANN models in the paper were the best of the considered models with the highest R value (>0.85). In addition, the number of nodes in the hidden layer was required to be minimal because increasing the number of nodes increases the processing time. All the algorithms of ANNs were composed in MATLAB with 0.50 training and test rates and included in the Levenberg–Marquardt algorithm (LMA). Also, Sigmoidal Function (SF) and Mean Squared Error (MSE) performance functions were employed in the algorithm.

## 3. Results

### 3.1. Analytical Approach Results

In the previous section, steel ball equivalence (SBE) was defined, and the effect of a steel ball (S) on the WA of RA was found to be approximately equal to 42 drum rotations (R) (Equation (5). In this section, the findings were examined for RAs sourced by low, medium, and high-strength CWs and supported by the experiments and literature (Table 7).

The results of the proposed equation and experimental results were compared with the results of the 400R-7S and 200R-12S combinations, and the combinations had 694 AC and 704 AC, respectively, and the WA of RA processed by the 400R-7S and 200R-12S combinations were 0.84% and 0.92%, respectively. The predicted WA of RA processed by 400R-7S and 200R-12S combinations were 0.96% and 0.87%, respectively. The difference between the experimental and predicted results for the 400R-7S and 200R-12S combinations was found very close to the experimental results.

In the literature, Babu et al. [29] used various R-S combinations of BMM to treat RA in their research, and the combinations employed included 200R-10S, 500R-10S and 700R-10S. The combinations were equal to 620 AC, 920 AC, and 1120 AC, respectively, according to Equation (5). The WA of RAs processed by 200R-10S, 500R-10S and 700R-10S were found to be 1.82%, 1.47%, and 1.39%, respectively [29] and the predicted values of WA of RA were calculated as 2.42%, 1.17%, 0.34%, respectively. Sui et al. [30] used RA with 5.7% WA and processed RA for 25 min (it equals 830 AC according to Equation (5) (Table 7)). Then, the WA of processed RA was found to be 1.60%. The predicted WA of processed RA was 1.13%, with a little difference. The predicted results of the literature showed that, although the difference values were high, when they were compared to the experimental results, the gradual improvement in the impact of BMM could be analytically obtained in Equation (5) and performed satisfactory results.

**Table 7:** Water absorption values of RAs.

Coarse RAs	Applied BMM Combination	%WA		Estimated %WA According to Equation (6)	AC
		Before application of BMM	After application of BMM		
RA sourced by low-strength waste (11–22 mm)	500R-10S	8.95	0.84	0.96	920
	300R-15S		0.92	0.87	930
RA sourced by medium-strength waste (4–11 mm)	400R-7S	4.21	1.63	2.14	694
	200R-12S		1.50	2.09	704
RA sourced by high-strength waste (4–11 mm)	500R-5S	3.52	1.28	1.73	710
	300R-16S		0.83	0.81	972
RA (8–16 mm) [30]	Only up to 25 min rotation is applied (BMM drum frequency ~33 rotations per min.)	5.7	1.60	1.13	830
RA (5–20 mm) [29]	200R-10S	4.16	1.82	2.42	620
	500R-10S		1.47	1.17	920
	700R-10S		1.39	0.34	1120

Not only the analytical equation derivation was proposed and compared with the test results and the literature data, but also the advantage of the proposed Equation (5) on AC was evaluated in terms of time-saving, equipment limitation and planning BMM impact. According to Equation (5), a steel ball (S) was equal to 42 drum rotations (R), and S can be preferred instead of 42 R. In BMM, the standard drum speed is 33 rotations/min, and thus the processing time can be decreased. Let us assume that a BMM application process with 400R-7S (694 AC) and 200R-12S (704 AC) would be considered to treat RA. The BMM application processes with 400R-7S (694 AC) and 200R-12S (704 AC) took 12 min and 6 min, respectively, and the time was reduced from 12 min to 6 min. In addition, the experimental results of WA of RA processed by 200R-12S and 400R-7S were 1.50% and 1.63%, respectively, and the predicted values of those were 2.09% and 2.14%, respectively (Table 7). The test results can be broadened for other RAs sourced by concrete wastes in many strength grades (low, medium and high grades), as given in Table 7.

Besides, the plan of BMM combination can be made under limited steel balls in terms of Equation (5). If the required number of steel balls is higher than the available steel balls, the drum rotations can be increased using the definition: Steel ball equivalence (SBE). Thus, BMM treatment can be made by adjusting the numbers of R and S in terms of SBE.

### 3.2. Artificial Intelligence (Artificial Neural Network Method) Results

This section presents the results of the mentioned artificial intelligence (Artificial Neural Network) analysis and its results. The WA of RA was estimated by ANN, and ANN model structures were designed differently for each analysis. The first ANN model included drum rotation–steel ball–water absorption; the second ANN model included Abrasion Coefficient–water, and the third one included rotation–steel ball–Abrasion Coefficient–water absorption. Accordingly, three analyses were performed on the parameters. ANN models were trained using the test data, and the train data selection in the analysis was random, but the rate of the training process can be given. The ANN models utilized 0.50 training and 0.50 testing rates. The Mean Squared Error (MSE) performance function was the performance indicator to assess the validity of the model. In addition, the predicted and test data were evaluated in terms of the Regression Coefficient (*R*). The summary of the results of the ANN models is presented in Table 8.

**Figure 6:**
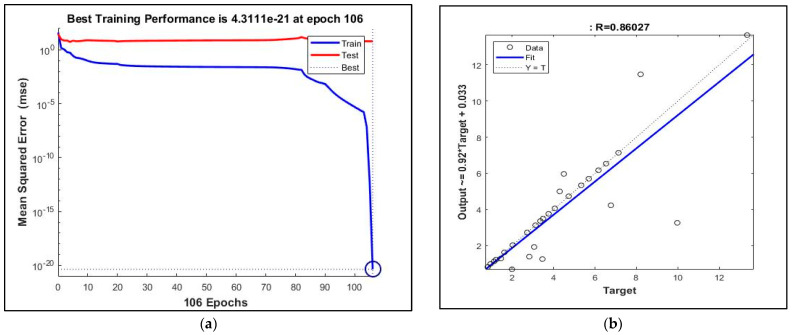
ANN results: (**a**) Training performance of artificial neuron network architecture for drum rotation-steel ball-water absorption. (**b**) The relation between predicted values of artificial neuron network architecture for drum rotation-steel ball-water absorption (*y*-axis) and experimental values (*x*-axis).

The training performance of ANN for drum rotation–steel ball–water absorption resulted in 106 epochs and R = 0.86027 (Figure 6). An ANN model prediction success can be read from a high R value (>0.75). The MSE value of the ANN model was 4.3111 × 10^−21^. The findings meant that the prediction was made with 106 epochs and a minimum square error of 4.3111 × 10^−21^, and the WA of RA was predicted with high accuracy by ANN, where the input parameters of the ANN were rotation and ball.

**Figure 7:**
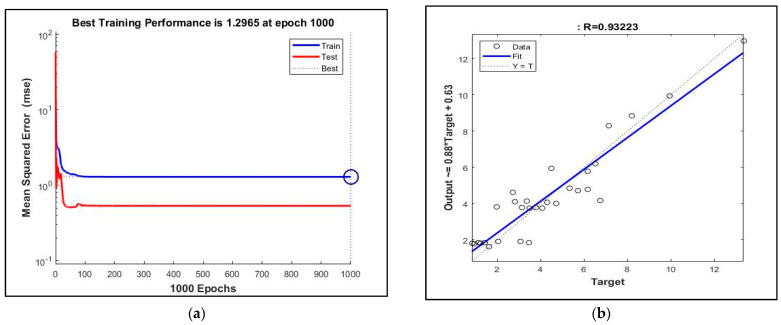
ANN results: (**a**) Training performance of artificial neuron network architecture for Abrasion Coefficient-water absorption. (**b**) The relation between predicted values of artificial neuron network architecture for Abrasion Coefficient-water absorption (*y*-axis) and experimental values (*x*-axis).

On the other hand, the training performance of ANN for Abrasion Coefficient-water absorption resulted in 1000 epochs and R = 0.93223 (Figure 7). The ANN model was successful in the estimation of WA of RA. However, when the results of ANN for drum rotation–steel ball–water absorption and ANN for Abrasion Coefficient-water absorption were compared, the number of epoch and MSE of the latter was higher than those of the former, while both ANN models had high *R* values (these were 0.86027 for the former and 0.93223 for the latter). In addition, the convergence problem was found for ANN for the Abrasion Coefficient–water absorption, and so the epoch and MSE were found to be high. Here, 1000 epochs was the default value in MATLAB if a convergence problem occurred.

**Figure 8:**
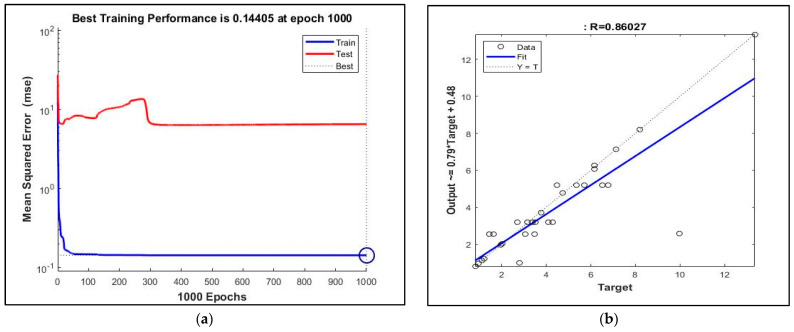
ANN results: (**a**) Training performance of artificial neuron network architecture for rotation-steel ball-Abrasion Coefficient-water absorption. (**b**) The relation between predicted values of artificial neuron network architecture for rotation-steel ball-Abrasion Coefficient-water absorption (*y*-axis) and experimental values (*x*-axis).

The convergence problem mentioned above was found for the ANN model composed for the rotation–steel ball–Abrasion Coefficient–water absorption, and the epoch and MSE were determined to be as high as ANN for the drum rotation–steel ball–water absorption. The epoch was asymptote to 1000, and the MSE was obtained with a value of 0.14405 (Figure 8). The *R* value, which measured the accuracy of the ANN model, was calculated to be 0.86027.

Table 8 presents the summary of the ANN structures and the obtained results for each ANN model. Accordingly, the input/hidden layer/output structures of the ANN models demonstrated satisfactory results with high R values (>0.85). WA of RA was estimated with high accuracy by ANN models, although the input parameters of the ANN models were different. Besides, the proposed AC approach was used in the second ANN model to predict the WA of RA, and the ANN had the highest R value (0.03223) compared to the others. As mentioned above in the Section 3.1, AC can be used as a key parameter to adjust the combination of BMM, and the findings of the current section supported the inference. Hence, the AC proposal, in terms of the ability of the equation to predict, showed its availability for both further research and use in industry.

In this paper, Equation (2) defined the linear relationship between the R-S and WA of RA, and ANN also defined the relationship between the R-S and WA of RA. Although the R values of the Equation (2) and ANN models were high (>0.75) and the predicted results of Equation (2) and ANN were meaningful, ANN was a more capable approach. ANN can be applied to both linear and non-linear problems to define the relationship between the input and output parameters [31].

### 3.3. Evaluations of Results

RA is a byproduct obtained as a result of grinding building demolition wastes in aggregate size (usually in the range of 0.125–32 mm). There is a high amount of waste concrete in the content of CDW, and in addition to this, it may contain other content, such as plaster, marble, or tile [27]. However, the use of CDW consisting of waste concrete as RA is more common, and its properties are better than others. Due to the presence of AOM in RA, the usability of RA in possible engineering applications is limited. For this reason, the increase in the quality of RA as a result of an improvement process (especially the decrease in water absorption value (<1%)) facilitates its use. The high water absorption value threatens the properties (especially the permanence) of the end product when RA is used. The RA usage constraint encountered in the construction industry creates a bias against RA. For this reason, it is considered to be an obstacle to its use. However, RA is an important recycled material for green buildings and green city concepts. Because these applications aim for and require creating the environment with recycled materials and sustainable technologies, the author thought that the results obtained within the scope of this article could reduce the biases on the use of RA. When the results obtained are evaluated, the water absorption value of around 8% can be adjusted at the desired level, and the necessary time and BMM combination for this process can be easily adjusted. On the other hand, there are aggregate standards, specifications and regulations required by the countries, and the BMM process to be applied in order to ensure compliance with these can be easily achieved. Thus, the restrictions and obstacles to the use of RA will be overcome. As a result, the use of RA in green-labeled buildings and environmentally friendly zero carbon emission cities will be easier, and the data/findings obtained will be able to find a response in the engineering industry.

## 4. Conclusions

In this paper, the gradual improvement effect of the Ball Mill Method (BMM) on recycled aggregate (RA) was investigated in terms of analytical and artificial intelligence approaches. In the analytical approach, equation derivations were made for the BMM gradual treatment effect, and new terms were proposed. In the artificial intelligence approach, an artificial neural network (ANN) was employed, and three ANN structures were constructed to observe the impact of the input parameters on the RA feature. As a result, the following were stated as conclusions:

- Analytical Approach: The BMM has two main instruments: Drum rotation (R) and steel ball (S). The effects of R and S on the water absorption (WA) of RA were different. The effect of R and S on the WA of RA can be formulated. Also, the S value can be defined in terms of the R value. S was equal to 42 R in terms of treatment impact (WA reduction). In this case, if R and S were considered in a single parameter, the “Abrasion Coefficient” (AC), WA of RA can be formulated and estimated with AC instead of R and S. At this point, the obtained equations and inferences were supported by both experimental data and literature and their accuracy was determined. On the other hand, the obtained findings facilitate BMM combination planning. Thus, it was possible to design a BMM combination considering processing time and limited equipment (i.e., steel balls). The most important result obtained with the analytically developed equations was the combination design of BMM. Thus, the gradual improvement effect of BMM can be applied in a controlled manner.
- Artificial Intelligence Approach: The prediction ability of the artificial intelligence approach (ANN) was found to be more accurate. The structure design of ANN may be a key factor in the results, and the accuracy of the results was high (R > 0.85). Thus, ANN was found to be a useful tool for the prediction of WA of RA processed by BMM.

To conclude, future and present requirements such as green buildings and zero carbon cities require “green” labeled requirements at many points, from design to materials, from manufacturing to management. These issues, which are a necessity for a viable, sustainable environment, will be shaped by the topics that are now put into practice. For this reason, in the above-mentioned issues, RA will need to be present as a byproduct and have sufficient properties. The subject discussed in this study and the findings obtained will be able to shed light on future generations at these points.

## Figures and Tables

**Table 1:** The initial properties of RAs [21].

RA	Size (mm)	Water absorption (%)
	Between 4.00 and 11.2	8.80
	Between 11.2 and 22.4	8.95

**Table 2:** The water absorption values of processed 4–11.2 mm RA after the application of BMM [21].

Water Absorption Test Results (%)							
		Number of Steel Balls					
		0	2	5	7	10	12
Number of drum rotations	100	5.85	5.67	5.94	5.46	5.07	4.05
	200	5.74	5.14	3.39	3.39	3.75	3.50
	300	4.07	4.30	3.55	3.42	2.18	1.40
	400	5.71	5.18	3.24	2.96	2.33	1.76
	500	4.87	3.56	2.82	2.36	2.60	2.57

**Table 3:** The water absorption values of processed 11.2–22.4 mm RA after the application of BMM [21].

Water Absorption Test Results (%)							
		Number of Steel Balls					
		0	2	5	7	10	12
Number of drum rotations	100	13.34	8.19	6.16	6.17	4.73	3.77
	200	7.14	6.52	2.71	3.37	3.50	1.63
	300	4.48	5.34	4.29	3.14	3.08	3.48
	400	5.71	6.77	4.08	9.96	1.45	1.24
	500	2.82	1.98	2.03	1.14	0.84	0.96

**Table 4:** The equations for 4–11.2 mm and 11.2–22.4 mm RAs.

WA = a × R^n^ + b × S^m^					
Definitions	a	n	b	m	Correlation Coefficient (*R^2^*)
Equation for 4–11.2 mm RA	4.5	0.333	−0.04	1	0.91
Equation for 11.2–22.4 mm RA	7.3	0.400	−0.05	1	0.88

**Table 5:** Exemplary combinations and estimated water absorption values for 4–11.2 mm RA.

Combination Details						
Group No	Sample No	*R_i_*	*S_i_*	Water Absorption (%)	Estimated Water Absorption (%)	$\frac{\left|R_{i}-R_{i+1}\right|}{\left|S_{i}-S_{i+1}\right|}$
1	1-1	300	5	3.55	4.17	29
	1-2	100	12	4.05	4.32	
2	2-1	500	2	3.56	4.30	67
	2-2	300	5	3.55	4.17	
3	3-1	200	7	3.39	4.31	60
	3-2	500	2	3.56	4.30	
4	4-1	300	5	3.55	4.17	50
	4-2	200	7	3.39	4.31	
5	5-1	400	2	5.18	4.68	33
	5-2	100	12	4.05	4.67	

*R_i_* drum rotation numbers, *S_i_* steel ball number.

**Table 6:** Exemplary combinations and estimated water absorption values for 11.2–22.4 mm RA.

**Combination Details**						
Group No	Sample No	*R_i_*	*S_i_*	Water Absorption (%)	Estimated Water Absorption (%)	$\frac{\left|R_{i}-R_{i+1}\right|}{\left|S_{i}-S_{i+1}\right|}$
1	1-1	200	7	3.37	4.72	50
	1-2	300	5	4.29	4.43	
2	2-1	200	7	3.37	4.72	50
	2-2	400	2	6.77	4.60	
3	3-1	500	7	1.14	2.31	67
	3-2	300	10	3.08	2.20	
4	4-1	500	2	1.98	4.99	36
	4-2	100	12	3.77	4.98	
5	5-1	100	12	3.77	4.98	30
	5-2	400	2	6.77	5.05	

*R_i_* drum rotation numbers, *S_i_* steel ball number.

**Table 8:** Summary of the results of the ANN models.

**ANN Structure**				
Notation	Input/Hidden Layer/Output	Epochs	R	MSE
R-S-WA	2-6-1	106	0.86027	4.3111 × 10^−21^
AC-WA	1-3-1	1000	0.93223	1.2965
R-S-AC-WA	3-6-1	1000	0.86027	0.14405

## Data Availability

The data presented in this study are available on request from the corresponding author.
